# Releasing Bioactive Compounds from Brown Seaweed with Novel Cold-Adapted Alginate Lyase and Alcalase

**DOI:** 10.3390/md21040208

**Published:** 2023-03-27

**Authors:** Jun Jiang, Zhengqiang Jiang, Qiaojuan Yan, Susu Han, Shaoqing Yang

**Affiliations:** 1 College of Food Science and Nutritional Engineering, China Agricultural University, No.17 Qinghua East Road, Haidian District, Beijing 100083, China; jiangjun@cau.edu.cn (J.J.);; 2 Key Laboratory of Food Bioengineering (China National Light Industry), College of Engineering, China Agricultural University, No.17 Qinghua East Road, Haidian District, Beijing 100083, China

**Keywords:** *Undaria pinnatifida*, alginate lyase, *Pichia pastoris*, alginate oligosaccharide, protein hydrolysaste

## Abstract

Seaweeds are considered to be third-generation renewable biomasses, the comprehensive utilization of which has drawn increasing attention in recent years. A novel cold-active alginate lyase (VfAly7) was identified from *Vibrio fortis* and biochemically characterized for brown seaweed utilization. The alginate lyase gene was high-level expressed in *Pichia pastoris*, with an enzyme yield of 560 U/mL and a protein content of 9.8 mg/mL by high-cell density fermentation. The recombinant enzyme was most active at 30 °C and pH 7.5, respectively. VfAly7 was a bifunctional alginate lyase with both poly-guluronate and poly-mannuronate hydrolysis activities. On the basis of VfAly7, a bioconversion strategy for the utilization of brown seaweed (*Undaria pinnatifida*) was developed. The obtained AOSs showed stronger prebiotic activity towards tested probiotics when compared to that of commercial fructooligosaccharides (FOSs), while the obtained protein hydrolysates displayed strong xanthine oxidase inhibitory activity with IC_50_ of 3.3 mg/mL. This study provided a novel alginate lyase tool as well as a biotransformation route for the utilization of seaweeds.

## 1. Introduction

Seaweeds, as promising third-generation renewable biomasses, are considered to have more advantages than plants from land, such as remarkable energy production, lower carbon-neutral emissions, and higher potentiality for sustainability, etc. [1]. Brown algae are the most abundant group of seaweeds, and they have gained increasing interests due to their huge annual production, high contents of proteins and polysaccharides, and other bioactive substances [2]. *Undaria pinnatifida* is a kind of brown seaweed which has been widely cultivated in Asian countries, including China, Korea, Japan and Indonesia [3]. Traditionally, *U. pinnatifida* are used as food directly, or as raw materials for the extractions of polysaccharides and proteins [4,5], but the utilization efficiency still remains low [6].

Alginate is the predominant structural polysaccharide in *U. pinnatifida*, with content highs up to 20–40% (*w*/*w*, total dry weight) [7]. Alginate oligosaccharides (AOSs) are produced from alginate, and mainly constitute α-L-guluronate (G) and β-D-mannuronate (M) units. AOSs have been confirmed to show significant physiological activities, such as prebiotic, antioxidant, antimicrobial, immunomodulatory, antidiabetic, antihypertensive and antitumor activities, etc. [8]. However, due the high content of intracellular alginate and its strong gel formation property, it is difficult to produce AOSs from brown seaweeds directly [9]. The traditional method for AOSs production includes two steps: (1) the chemical extraction of alginate, and (2) the acid hydrolysis of alginate. However, some shortcomings exist in the procedure, such as environmental pollution, low extraction efficiency, and unstable product composition [10,11]. In addition to AOSs production, algae are also an important protein source and have been used to produce various bioactive protein hydrolysates/peptides, such as antidiabetic, antihypertensive and antioxidant protein hydrolysates/peptides [12,13]. Xanthine oxidase (XOD) is the key enzyme that catalyzes the production of uric acid from hypoxanthine and xanthine. Previous studies have suggested that the production of uric acid in the human body can be reduced by inhibiting the activity of XOD, and the purpose of preventing hyperuricemia can be achieved [14]. Therefore, it is of great value to explore effective XOD inhibitors to prevent hyperuricemia. However, no protein hydrolysates with xanthine oxidase (XOD) inhibitory activities have been identified from *U. pinnatifida* yet.

Alginate lyases can depolymerize alginate into AOSs by cleaving glycosidic bonds through a β-elimination reaction [15]. The AOSs produced by alginate lyase had a double bond between C4 and C5 at the non-reducing end, which showed higher biological activities [2]. According to the protein sequence similarity, alginate lyases are distributed in the polysaccharide lyase (PL) families 5, 6, 7, 8, 14, 15, 17, 18, 31, 32, 34, 36, 39 and 41 in the Carbohydrate-Active Enzymes (CAZy) database (http://www.cazy.org/, accessed on 16 March 2022). Recently, cold-active alginate lyases such as TsAly6A from *Thalassomonas* sp. LD5 [16], rA9mT from *Vibrio* sp. JAM-A9m [17] and AlyS02 from *Flavobacterium* sp. [18] have been drawing increasing attention from an in industrial point of view. It is worth noting that cold-adapted alginate lyases make biocatalysis at low temperatures possible. The use of alginate lyases with high activity and catalytic efficiency at low temperature can reduce energy consumption, prevent the reproduction of harmful microorganisms and terminate reaction processes by raising the temperature slightly [19]. However, up to now, the limited reported cold-adapted alginate lyases could hardly satisfy the commercial requirements due to their weak stability and low yields [16,20].

In this investigation, a novel alginate lyase from *Vibrio fortis* was high-level expressed in *P. pastoris* and characterized for the utilization of brown seaweed (*U. pinnatifida*). On the basis of the novel cold-active alginate lyase and commercial protease, an efficient strategy for the productions of AOSs and bioactive protein hydrolysates from *U. pinnatifida* was developed. The prebiotic activity of AOSs and XODs inhibitory activity of the protein hydrolysates were further evaluated.

## 2. Results and Discussion

### 2.1. Expression of the Alginate Lyase Gene in P. pastoris

**Figure 1:**
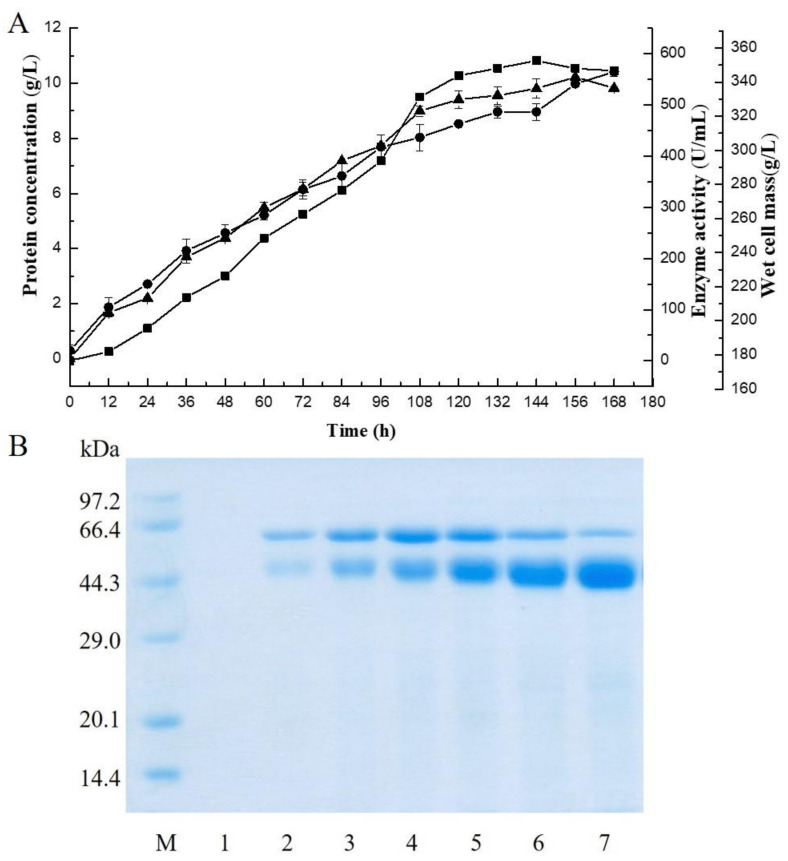
Time-course of the VfAly7 expression in *P. pastoris* (**A**) and the extracellular protein analysis during high–cell density fermentation (**B**). (**A**) Enzyme activity (■), protein concentration (●) and cell mass (▲) of culture broth. The samples were taken every 12 h. (**B**) Lane M, low molecular weight protein standards; lanes 1–7, samples withdrawn at 0, 24, 48, 72, 96, 120 and 144 h, respectively, after methanol induction.

An alginate lyase gene (*VfAly7*) from *V. fortis* was cloned and expressed in *P. pastoris*. The predicted molecular mass and *p*I of the mature protein were predicted to be 54 kDa and 4.81, respectively. The coding sequence of the gene had an N-terminal CBM32 domain, a C-terminal PL family 7 domain, and a linker connecting the CBM32 and PL7 domains. It shared the highest identity of 82% with the PL family 7 alginate lyase (Alg7A) from a marine bacterium *Vibrio* sp. W13 (Figure S1) [21]. Thus, VfAly7 should be a novel alginate lyase in PL family 7. A transformant displaying the highest enzyme activity was subjected to high–cell density fermentation in a 5–L fermenter, and the highest extracellular alginate lyase activity of 560 U/mL, with a protein concentration of 9.8 mg/mL and a wet cell mass of 380 g/L, was obtained after 144 h of cultivation (Figure 1A). SDS-PAGE analysis suggested that the recombinant alginate lyase (VfAly7) was the major protein in the culture broth during the fermentation process, accounting for >90% of total proteins (Figure 1B).

Enzymatic conversion is a promising method for the utilization of brown algae, e.g., AOSs and bioactive protein hydrolysate/peptide productions, in which specific alginate lyases play key role [22]. So far, though several alginate lyases suitable for AOSs production from brown algae have been identified from different sources, the enzyme yields remain low, and could hardly meet the industrial production requirement [19]. For instance, the alginate lyase gene (*paAlgL*) from *Pseudomonas aeruginosa* was overexpressed in *P. pastoris* with an enzyme activity of 21 U/mL [23]. The alginate lyase cAlyM from *Microbulbifer* sp. Q7 and its thermostable mutant 102C300C were expressed in *P. pastoris*, and the extracellular enzyme activities of 33.82 U/mL and 33.87 U/mL were obtained, respectively [24]. In this study, the alginate lyase gene (VfAly7) was expressed in *P. pastoris* and the highest yield of 560 U/mL (9.8 mg/mL) was obtained, which was only next to that of the alginate lyase rSAGL (226.4 µg/mL, 915.5 U/mL) from *Flavobacterium* sp. H63 expressed in *P. pastoris* [25]. The good yield may make VfAly7 a potential candidate for the industrial production of AOSs.

### 2.2. Biochemical Characterization of VfAly7

**Figure 2:**
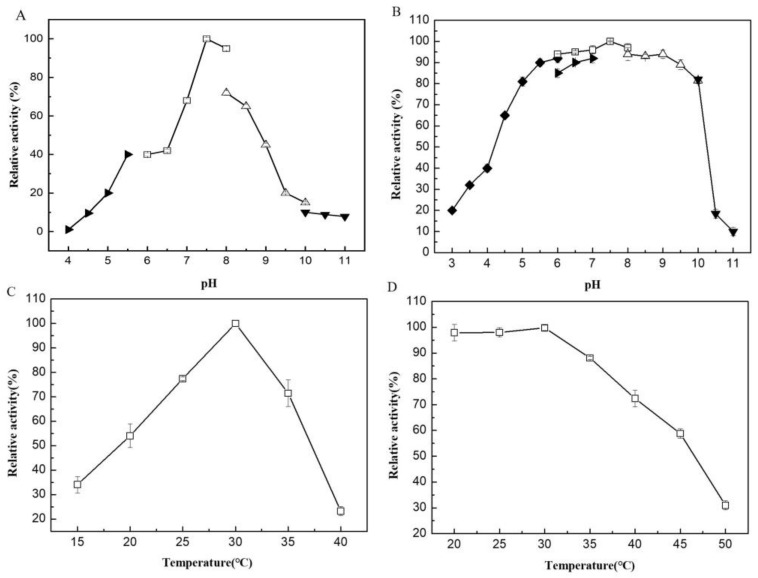
Optimal pH (**A**), pH stability (**B**), optimal temperature (**C**) and thermostability (**D**) of VfAly7. The buffers used are phosphate buffer (□, pH 6.0–8.0), citrate buffer (◆, pH 3.0–6.0), CAPS buffer (▼, pH 10.0–11.0), CHES buffer (△, pH 8.0–10.0) and MES buffer (►, pH 4.0–7.0). The optimal pH and pH stability of the purified VfAly7 were determined at 30 °C for 10 min. The optimal temperature and thermal stability were estimated in 50 mM phosphate buffer pH 7.5 for 10 min.

The recombinant enzyme was purified to homogeneity with a specific activity of 71.5 U/mg and a recovery yield of 72%. The purified VfAly7 migrated as a single band on SDS-PAGE gel with an estimated molecular mass of about 44 kDa (Figure S2). VfAly7 had an optimal pH of 7.5 in 50 mM phosphate buffer (Figure 2A), and it was stable within the pH range of 5.0–10.0 (Figure 2B). VfAly7 was most active at 30 °C (Figure 2C), and it was stable at temperatures ≤ 35 °C as ≥80% of its initial activity was retained after incubation at these temperatures for 30 min (Figure 2D).

Generally, the cold-adapted alginate lyases exhibit maximal activities at temperatures ≤ 35 °C, and retain ≥ 50% of their maximum activity at 20 °C [15]. VfAly7 was a cold-adapted alginate with an optimal temperature of 30 °C, which is similar to that of several other cold-adapted alginate lyases, having optimal temperatures in the range of 20–35 °C [16,17,18]. Though thermostable enzymes are unusually preferred from an industrial point of view, cold-adapted alginate lyases have their own advantages [22]. For example, the reactions performed at lower temperatures can avoid the denaturing of some unstable bioactive substances during the conversion process. In addition, the relatively low reaction temperatures can significantly reduce the consumption of energy [19]. VfAly7 showed a broad pH stability range of 5.0–10.0, which is wider than that of most other reported cold-adapted alginate lyases, such as A9m from *Vibro* sp. (pH 7.0–10.0) [17], AlyS02 from *Flavobacterium* sp. (pH 6.0–9.0) [18] and TsAly7B from *Thalassomonas* sp. (pH 7.3–8.6) [20]. Therefore, alginate lyases VfAly7 with unique properties of low optimal temperature and wide pH stable range may have great potential in industrial applications.

### 2.3. Substrate Specificity and Hydrolysis Property of VfAly7

**Figure 3:**
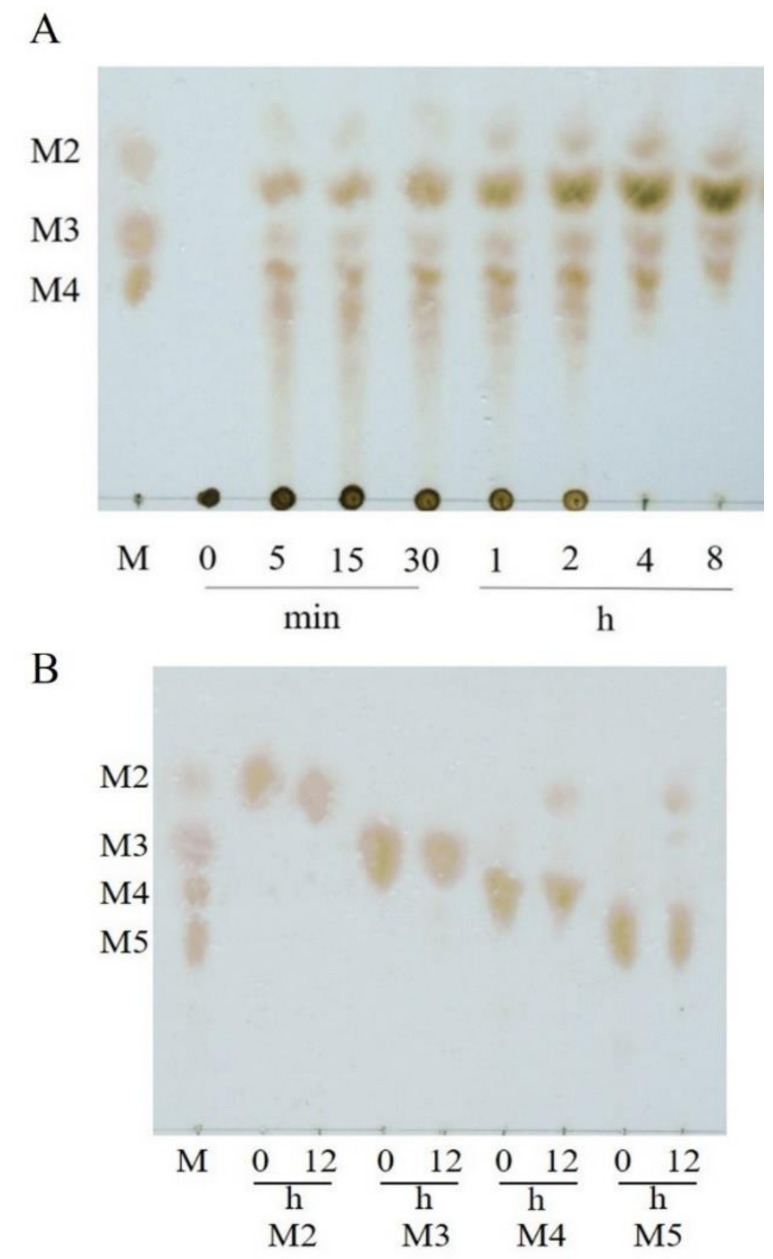
TLC analysis of hydrolysis products of alginate sodium (**A**) and mannuronate oligosaccharides (**B**) by VfAly7. The reactions were performed at 30 °C in 50 mM phosphate buffer pH 7.5, and the samples were withdrawn at different times. M2–M5 represent the mannuronate oligosaccharides with DPs 2–5, respectively. Lane M: standard mannuronic acid sodium salts.

VfAly7 exhibited the highest specific activity towards sodium alginate (71.5 U/mg), followed by poly MG substrate (68.3 U/mg), poly M block substrate (47.2 U/mg) and poly G block substrate (34.3 U/mg) (Table S1), suggesting that it is a bifunctional alginate lyase. VfAly7 hydrolyzed sodium alginate to yield mainly oligosaccharides with high DPs in the initial hydrolysis stage (0–30 min), and these intermediates were further converted to oligosaccharides with low DPs with the extension of the incubation time (Figure 3A), exhibiting a typical action manner of endo-type alginate lyases. VfAly7 catalyzed the hydrolysis of M4 to release mainly disaccharides, and hydrolyzed M5 to release disaccharides and trisaccharides, but could hardly hydrolyze M2 and M3 (Figure 3B).

Substrate specificity is an important property of endo-type alginate lyases for AOSs production from brown seaweed, as the activity of alginate lyases with substrate preference might be reduced by the substrate blocks in the sodium alginate, thus affecting the degradation rate and composition of products [19]. Most of endo-type alginate lyases are specific to M block or G block, while others showing specificity towards both M and G blocks are called bifunctional enzymes. For example, alginate lyase ALG-5 appeared to be a poly-guluronate lyase preferring to degrade poly–G block [26]. AlgA from *Bacillus* sp. Alg07 was a poly M-specific alginate lyase exhibiting high affinity towards poly M [27]. Similar to two other reported bifunctional alginate lyases [19,21], VfAly7 exhibited almost equal activities towards poly M and poly G, the properties of which may overcome the limitations of available various substrate sources and facilitate the production process. The major hydrolysis products of alginate by VfAly7 were AOSs with DPs 2–4, indicating that VfAly7 might be a potential tool for the production of AOSs with low molecular weight.

### 2.4. Production of AOSs and Their Prebiotic Activity

**Figure 4:**
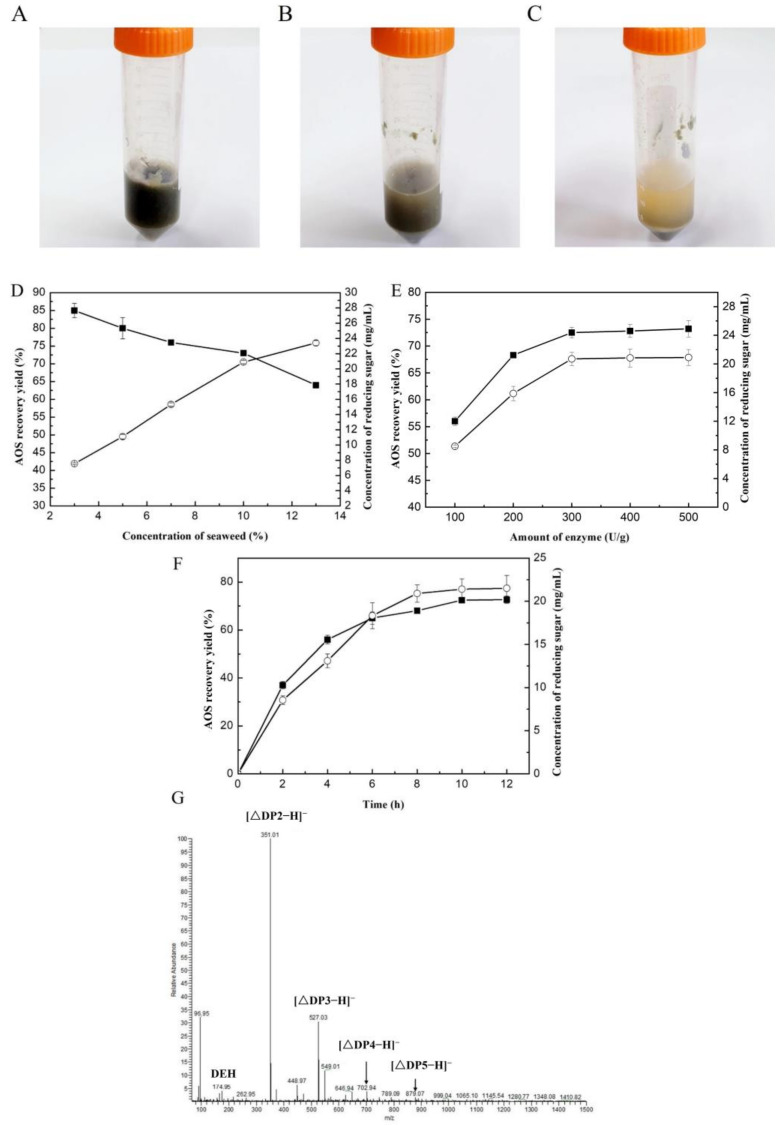
Enzymatic hydrolysis and optimizations of AOSs production from *U. pinnatifida*. The morphology of *U. pinnatifida* hydrolyzed by VfAly7 for 0 (**A**), 2 (**B**) and 8 h (**C**), respectively. Effect of substrate content (**D**), enzyme dosage (**E**) and hydrolysis time (**F**) on the production of AOSs, and composition analysis of obtained AOSs (**G**). The reactions were performed at 30 °C in 50 mM phosphate buffer pH 7.5. Enzyme concentration and duration were 400 U/g and 12 h in Figure 4D; substrate concentration and duration were 10% and 12 h in Figure 4E; substrate concentration and enzyme concentration were 10% and 300 U/g in Figure 4F. (■) AOSs recovery, (○) AOSs content.

**Figure 5:**
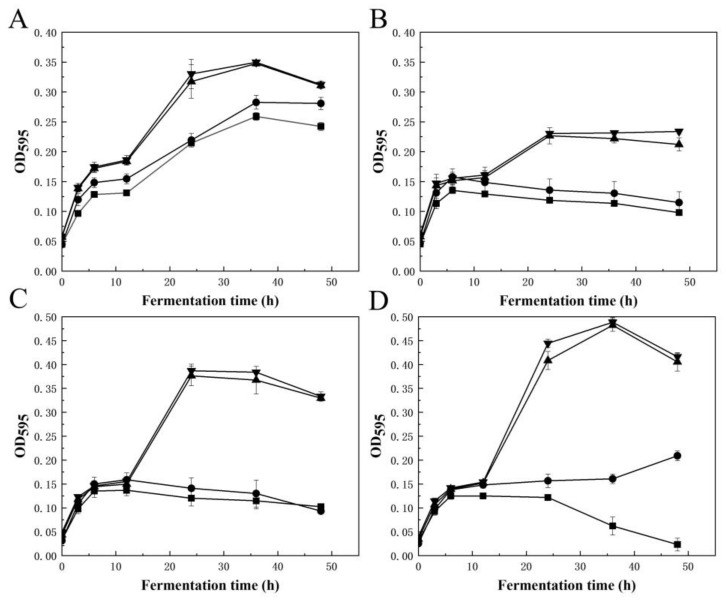
Growth profiles of *Lactobacillus brevis* NRRL B-4527 (**A**), *Lactobacillus casei* subsp. casei AS 1.2435 (**B**), *Bifidobacterium adolescentis* ATCC 15703 (**C**), *Bifidobacterium longum* NRRL B-41409 (**D**) using 1% (*w*/*v*) of AOSs (▲) from *U. pinnatifida* or sodium alginate (▼), 1% (*w*/*v*) of FOSs (●) as sole carbon source or without sugar (■) at 37 °C for 48 h in an anaerobic condition by mineral oil. AOSs from sodium alginate were prepared with 2.0 g sodium alginate and 100 U VfAly7 at 30 °C for 12 h. Growth curves of the strains were constructed by the OD_595_ values of fermentation medium using an automated microplate reader.

VfAly7 showed strong degradation ability on the raw material of *U. pinnatifida* and converted them into fragments and soluble substances in 8 h (Figure 4A–C). The substrate content and enzyme dosage for AOSs production were then optimized. With the increasing of the substrate content from 3% to 12%, the content of released reducing sugar increased from about 8.0 mg/mL to 23.5 mg/mL, while the AOSs conversion ratio reduced from 85% to 65% (Figure 4D). Taking into account yield and conversion ratio, 10% was chosen as the best substrate content for AOSs production, where a relatively high AOSs content of 21.0 mg/mL with a conversion ratio of 72.9% was obtained (Figure 4D). Subsequently, the enzyme dosage was optimized to be 300.0 U/g, where the reducing sugar of 20.9 mg/mL with AOSs conversion ratio of 73.0% was obtained (Figure 4E). In order to further confirm the composition of the oligosaccharides, the end products were subjected to negative-ion ESI-MS. The results indicated that the hydrolysis products were AOSs with DPs 2–5 (Figure 4G). Prebiotic activity analysis suggested that AOSs from *U. pinnatifida* and sodium alginate exhibited similar or strong prebiotic activity, as the growth of the tested *Lactobacillus* and *Bifidobacteria* strains was all significantly promoted when AOSs was used as the sole carbon energy source (Figure 5). The highest OD_595_ values of *L. casei* subsp. casei AS 1.2435 and *B. adolescentis* ATCC 15703 supplied with AOSs from *U. pinnatifida* reached 0.28 and 0.38, respectively, at 24 h (Figure 5B,C), which was about 1.75 and 2.24 times higher than that of the positive control supplemented with commercial FOSs, while those of *L. brevis* NRRL B-4527 and *B. longum* NRRL B-41409 reached high up to 0.35 and 0.48, respectively, at around 36 h (Figure 5A,D), which was about 1.25 and 3.0 times higher than that of the positive control supplemented with commercial FOSs.

Until now, no report has been available for AOSs production from *U. pinnatifida*. However, several attempts have been performed for AOSs production from other species of brown seaweeds [8,28]. For example, a combined strategy of enzymatic hydrolysis and selective fermentation was developed for AOSs production from brown seaweed *Saccharina japonica*. After 72 h of cultivation, glucose and mannitol derived from the initial hydrolysis of *L. japonica* were completely consumed, with AOSs yield of 91.7% [22]. It has been reported that the AOSs prepared by enzymatic and fermentation methods displayed different prebiotic effects on intestinal microbiota and biological activities when compared to those produced by physical and chemical methods, due to the unsaturated end structures and different DPs of the products [8,29]. So far, many biological activities of AOSs have been reported. Wang et al. [30] reported that AOSs exhibited stronger stimulation effects on the growths of *Bifidobacterium bifidum* ATCC 29521 and *Bifidobacterium longum* SW 27001 when compared to commercial prebiotic FOSs. In this study, the AOSs from brown seaweed (*U. pinnatifida*) showed significant growth promotion activity towards all the tested prebiotic strains, and the activity was much higher than that of commercial FOSs. Therefore, AOSs may be beneficial for human health by acting as prebiotics.

### 2.5. Production of Algal Protein Hydrolysate and Its XOD Inhibitory Activity

**Figure 6:**
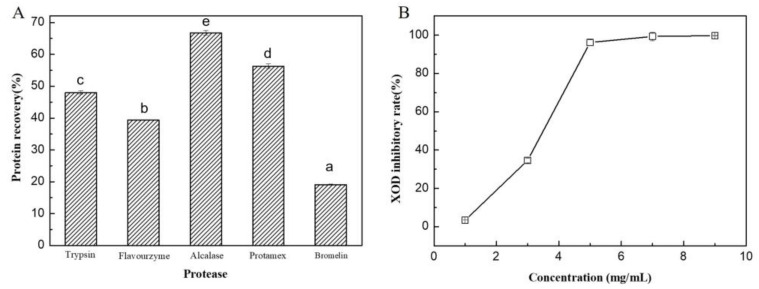
Recovery of algal protein hydrolysates by different proteases (**A**) and XOD inhibitory activity of the hydrolysates (**B**). The hydrolysis reactions were performed at the optimal temperatures and pHs of the correspondent proteases for 12 h. Values with different letters (a–e) indicate a significant difference (*p* < 0.05).

The content of crude protein in the brown seaweed raw material was determined to be 15.3% (*w*/*w*), and the content in the separated solid residues was enriched to 29.3% (*w*/*w*) after enzymatic hydrolysis, due to the release of other components (i.e., alginate, glucan, mannitol, etc.) into liquid solution. Overall, about 90.0% of proteins were recovered in solid residues from the brown seaweed raw material. Among the tested proteases, alcalase was found to be the most suitable enzyme for brown seaweed protein hydrolysis, and the highest protein conversion ratio of 61.4% was obtained (Figure 6A). Alcalase was used to calculate the XOD inhibitory activity of the hydrolysates, and the IC_50_ value of the hydrolysate for the XOD inhibitory rate was determined to be 3.3 mg/mL (Figure 6B).

The utilization of seaweed proteins has drawn much attention with the increase in global demand for edible proteins in recent years [4]. However, the process is difficult as the digestibility and the extractability of seaweed proteins are inhibited by the profusion of polyphenols and strong cell walls rich in polysaccharides [31]. In particular, the existence of alginates could significantly increase the viscosity of the seaweed suspension, thus reducing the protein extraction or hydrolysis efficiency [32]. Hence, it is quite necessary to avoid the obstacle by degrading alginates prior to protein extraction and hydrolysis. In this study, most of alginates in seaweeds were degraded during the production of AOSs, which facilitated protein extraction and hydrolysis.

Recently, the identification and production of food-derived bioactive protein hydrolysates/peptides with healthy activities and minimal side effects, such as XOD inhibitory activity from different protein sources, has gradually become the research focus instead of protein extraction. For example, a bioactive milk peptide (YLDNY) and two walnut protein peptides (WPPKN and ADIYTE) were found to show XOD inhibitory activity [33,34]. Moreover, some proteins from marine fishes, such as bonito, tuna and shark, have also been found to be good sources for the production of XOD inhibitory peptides [35]. In addition to marine animal proteins, marine plant proteins are also good sources for the production of bioactive protein hydrolysates/peptides. Recently, the edible seaweed *U. Pinnatifida* has been used to produce antioxidant and antihypertensive protein hydrolysates [4]. However, no study has been performed on the production of protein hydrolysates with XOD inhibition activity from this kind of seaweed. In this study, the bioactive protein hydrolysates with XOD inhibitory activity were produced by enzymatic hydrolysis of proteins from *U. Pinnatifida*, and the XOD inhibitory rate (IC_50_ = 3.3 mg/mL) was much lower than that of extracts obtained from other raw materials, such as that from *Skipjack tuna* (IC_50_ = 7.2 mg/mL) [14] and walnut meal (peptide WPPKN, IC_50_ = 17.8 mg/mL, peptide ADIYTE, IC_50_ = 19.0 mg/mL) [33]. Hence, the hydrolysates of brown seaweed may have application potential in the food industry as a new type of functional food material.

### 2.6. Enzymatic Approach for the Production of Bioactive Compounds from Brown Seaweed

A flow diagram of the enzymatic processing for the production of bioactive compounds from *U. pinnatifida* is shown in Figure S3. The content of alginic acid in the brown alginate was about 21.0 g/100.0 g. After hydrolysis and separation procedures, the captured AOSs was about 17.4 g/100.0 g substrate, and the concentration of uronic acid in the AOSs was calculated to be 88.2% (*w*/*w*). Therefore, the AOSs recovery yield was about 73.0% in the procedure. The separated precipitates after hydrolysis were treated with alcalase, and the majority of the proteins (61.4%, *w*/*w*) were converted into soluble protein hydrolysates with high XOD inhibitory activity.

More and more marine plant sources and relevant by-products are being transformed and utilized as valuable functional foods or additives with the development of marine bioprocess technology [36]. In this study, a new efficient process for the production of AOSs (especially for low molecular mass AOSs) from *U. pinnatifida* using a novel recombinant alginate lyase VfAly7 was developed, which did not require an intermediate step to separate alginate and can be completed in 10 h. Moreover, the process facilitated the subsequent bioconversion of proteins to bioactive hydrolysates. Overall, the process is much simpler and more efficient than the traditional methods. Therefore, the enzymatic approach may be a suitable solution for the bio-refining of brown seaweeds.

## 3. Materials and Methods

### 3.1. Materials

*E. coli* DH5α was obtained from TaKaRa Corporation (Dalian, China). The heterologous protein expression host of *P. pastoris* GS115 and vector pPIC9K were bought from Invitrogen (Carlsbad, CA, USA). The probiotic strains, including *Lactobacillus brevis* NRRL B-4527, *Lactobacillus casei* subsp. casei AS 1.2435, *Bifidobacterium longum* NRRL B-41409 and *Bifidobacterium adolescentis* ATCC 15703, were provided by the China Center of Industrial Culture Collection (CICC).

Sodium alginate was obtained from GUOYAO Co., Ltd. (Shanghai, China). Saturated mannuronate acid sodium salts standards, poly M block and poly G block (purity ≥ 95%) were the products of Qingdao BZ Oligo Biotech Co., Ltd. (Qingdao, China). Poly MG was prepared as in the method described previously [37]. Fructooligosaccharides (FOSs, Raftilose P95) with degrees of polymerization (DPs) 2–7 were obtained from Orafti Group (Tienen, Belgium). Trypsin, flavourzyme, alcalase, protamex and bromelin were obtained from LongDa Biotechnology Co. Ltd. (Shandong, China).

### 3.2. Gene Cloning and Expression

An alginate lyase gene (*VfAly7*) was found at the NCBI server and synthesized by Shanghai Generay Biotech. The gene was amplified using the primers of VfAly7F (5′GAATTCCCAGTTGTTGAACAATTTCCTAAC3′) and VfAly7R (5′GCGGCCGCTCAACCTTGAAATTCTCCGTAAGTAG3′). The amplified PCR products were cloned into the pPIC9K vector, and then transformed into *E. coli* DH5α for plasmid propagation. The recombinant plasmid pPIC9K-VfAly7 was linearized and transformed into *P. pastoris* GS115. The colonies showing G418 resistance were selected for the expression of alginate lyase under the Invitrogen’s guidelines.

The recombinant strain showing the highest enzyme activity was selected and inoculated in a 5 L fermenter, according to the *Pichia* Fermentation Process Guidelines (Version B, 053002, Invitrogen). Samples were withdrawn at different times, and the alginate lyase activity, protein concentration, and wet cell mass were monitored.

### 3.3. Enzyme Activity Determination

Alginate lyase activity was determined following the method of Meng et al. [38]. Namely, 0.1 mL of suitably diluted enzyme solution was added in 0.9 mL of sodium alginate solution (0.3%, *w*/*v*) in 50 mM phosphate buffer pH 7.5, and incubated at 30 °C for 10 min. After the termination of the reaction by heating with boiled water for 10 min, the content of released reducing sugars was determined by the 3,5-dinitrosalicylic acid (DNS) method [39]. One unit (U) of alginate lyase activity was defined as the amount of enzyme liberating 1 μmol glucose equivalent reducing sugars per minute under the assay conditions.

The protein content was determined by the method of Lowry et al. [40] using bovine serum albumin as the standard.

### 3.4. Purification and Biochemical Characterization of VfAly7

The crude enzyme was collected by centrifuging of the culture broth at 10,000× *g* for 10 min. After dialyzed against phosphate buffer (pH 7.0) at 4 °C for 12 h, the crude enzyme was load onto a pre-equilibrated Q-Sepharose column (10 × 1 cm, GE Healthcare) at a flow rate of 0.5 mL/min. The impurities were removed by six column volumes of phosphate buffer (pH 7.0), and the bound alginate lyases (VfAly7) were eluted with a 100–500 mM linear gradient of NaCl in phosphate buffer (pH 7.0). The fractions with alginate lyase activity were collected and checked for purity by SDS-PAGE.

The optimal pH of the purified VfAly7 was determined by measuring the enzyme activity in 50 mM various buffers within pH 4.0–11.0 at 30 °C. The buffers used were citrate buffer (pH 3.0–6.0), MES buffer (pH 4.0–7.0), phosphate buffer (pH 6.0–8.0), CHES buffer (pH 8.0–10.0) and CAPS buffer (pH 10.0–11.0). For pH stability determination, VfAly7 was incubated in different buffers mentioned above at 30 °C for 30 min and the retained activities were then measured using the standard enzyme assay. The optimal temperature was estimated by measuring the enzyme activity at different temperatures (15–40 °C) in 50 mM phosphate buffer pH 7.5. For the thermal stability determination, the enzyme was incubated at different temperatures (20–50 °C) for 30 min in 50 mM phosphate buffer pH 7.5, and the retained activities were then measured by the standard assay.

### 3.5. Substrate Specificity and Hydrolysis Properties of VfAly7

The substrate specificity of VfAly7 was analyzed by determining the enzyme activity in 50 mM phosphate buffer pH 7.5 at 30 °C by the standard enzyme assay using 1% (*w*/*v*) of different substrates, including sodium alginate, poly M, poly G, poly MG, chitin, agarose, cellulose, mannan and CMC.

To determine the action mode of VfAly7, the hydrolysis products of different substrates including sodium alginate and saturated mannuronic acid sodium salts with DPs 2–6 (M2–M6) were analyzed by thin-layer chromatography (TLC). A total of 1 U/mL of purified enzyme was added into 1 mL of different substrates (1%, *w*/*v*) in 50 mM phosphate buffer pH 7.5, and incubated at 30 °C for 12 h. Samples taken at different times were immediately boiled for 5 min, centrifuged at 12,000× *g* for 10 min, and then subjected to TLC analysis on a silica gel 60 TLC plate (Merck, Darmstadt, Germany). For TLC analysis, a solvent system consisting of n-butanol: formic acid: water [2:1:1 (*v*/*v*/*v*)] was used, and the products were visualized by immersing the plate in H_2_SO_4_, solution (5%, *v*/*v*), followed by heating in an oven for few seconds.

### 3.6. Degradation of Brown Seaweed by VfAly7

Fresh brown seaweed was washed thoroughly with tap water, dried at 65 °C for 4 h, and then crushed and filtered through a 60-mesh sieve. The seaweed powder was suspended in distilled water and hydrolyzed by VfAly7 at 30 °C with constant agitation (200 rpm). The substrate content (3%, 5%, 7%, 10% and 13%, *w*/*v*), enzyme dosage (100, 200, 300, 400 and 500 U/g) and hydrolysis time (2, 4, 6, 8, 10 and 12 h) were optimized one by one. The reaction was stopped by heating at 80 °C for 10 min. The mixture was centrifuged at 10,000× *g* for 10 min, and the supernatant was collected for AOSs analysis, while the precipitate was collected and dried for protein hydrolysis.

### 3.7. Recovery of AOSs from Brown Seaweed by VfAly7

High molecular mass soluble polysaccharides in the hydrolysate were precipitated by the addition of an equal volume of ethanol. After storage at 4 °C for 12 h, the precipitate was removed by centrifugation at 5000× *g* for 10 min and the supernatant was concentrated by rotary evaporation at 50 °C for AOSs recovery. AOSs were precipitated by the addition of five volumes of ethanol. After centrifugation at 5000× *g* for 15 min, the precipitate was collected, freeze-dried and analyzed.

The content of uronic acids in AOSs was determined by the m-hydroxyldiphenyl method [41]. The DPs and precise molecular masses of the major ingredients in the AOSs mixture were analyzed by TLC and electrospray ionization mass spectrometry (ESI-MS), respectively. The ESI-MS was performed in a negative mode with following parameters: ion source voltage of 4.0 kV, capillary temperature of 275–300 °C, tube lens of 120 V, sheath gas of 45 arbitrary units (AU), and mass range of 50–1500 *m*/*z*. The AOSs recovery yield was estimated by the method of Li et al. [22] using the following Equation (1):

AOS recovery yield (%) = W1/W2 × 100,(1)

where W1 and W2 represent the uronic acid contents in the recovered AOSs and original seaweed sample, respectively.

### 3.8. Prebiotic Activity Assay of AOSs

The effect of AOSs on the growth of probiotic strains was evaluated according to the method of Liu et al. [42]. Briefly, the selected probiotic strains were cultured in a 96–well plate (costar 3599) at 37 °C in an anaerobic condition sealed by mineral oil using 1% (*w*/*v*) of AOSs or FOSs as the sole carbon source, or without carbon source. For the cultivation of *Bifidobacterium* strains, 0.05% (*w*/*v*) of L-cysteine hydrochloride was additionally added to the medium. The tested strains included *L. brevis* NRRL B-4527, *L. casei* subsp. casei AS 1.2435, *B. longum* NRRL B-41409 and *B. adolescentis* ATCC 15703. The absorbance of the culture broth at 595 nm was recorded at different times.

### 3.9. Recovery of Protein Hydrolysates with XOD Inhibitory Activity

The dried precipitation powder was dissolved in distilled water at a proportion of 5% (*w*/*v*), and the pH was adjusted to the optimal pH of the correspondent proteases used. Then, 1% (*w*/*w*) of different proteases, including trypsin (pH 8.0, 37 °C), flavourzyme (pH 7.0, 53 °C), alcalase (pH 8.5, 60 °C), protamex (pH 8.0, 55 °C) and bromelin (pH 8.0, 55 °C) were added and the mixtures were incubated at their optimal temperatures and pHs for 12 h, separately. After the reaction was terminated by heating at 85 °C for 15 min, the protein content was determined by the Lowry method [40]. The hydrolysate was collected for XOD inhibitory activity assay.

### 3.10. XOD Inhibitory Activity Assay

XOD inhibitory activity of the hydrolysate was determined according to the method of Zhong et al. [14] with minor modifications. Firstly, 50 μL of hydrolysate was added to 150 μL of XOD solution (0.05 U/mL) in phosphate buffer (50 mM, pH 7.5) and pre-incubated at 37 °C for 5 min. Then, 150 μL of xanthine (150 μM) was added to the mixture and further incubated at 37 °C for 60 min. Finally, the reaction was terminated by the addition of 100 μL of 1 M HCl. The absorbance of the reaction mixture at 290 nm was measured. The XOD inhibitory rate (%) was calculated following Equation (2).

(2) $$\mathrm{XOD}\;\mathrm{inhibitory}\;\mathrm{rate}\;(\%)=(1–\;\frac{A1-A2}{A3-A4})\times 100,$$

where *A*1, *A*2, *A*3 and *A*4 are the absorbance values of sample and XOD mixture, sample alone, buffer and XOD mixture, and used buffer alone, respectively. The IC_50_ of XOD inhibition was calculated.

## 4. Conclusions

A novel cold-active bifunctional alginate lyase from *V. fortis* was identified and biochemically characterized for the utilization of brown seaweed. The enzyme was high-level expressed in *P. pastoris*, with an enzyme production of 560 U/mL. The enzyme was most active at 30 °C and pH 7.5. On the basis of the newly found enzyme, an efficient, low-cost and environmental friendly strategy for the recovery of alginates and proteins in brown seaweed (*U. pinnatifida*) was developed. By using the strategy, AOSs with probiotic functions and bioactive protein hydrolysates with XOD inhibitory activity were obtained with conversion ratios of 73.0% and 61.4%, respectively. The study may provide an efficient and green routine for the utilization of renewable marine algae.

## Data Availability

Not applicable.

## Supplementary Materials

The following supporting information can be downloaded at: https://www.mdpi.com/article/10.3390/md21040208/s1, Table S1: Substrate specificity of VfAly7; Figure S1: Multiple sequence alignment of VfAly7 and other well-characterized alginate lyases from PL family 7. Identical residues are shown in white on a red background, and conserved amino acid regions are marked in the green boxes; Figure S2: Purification of the recombinant VfAly7 expressed in *P. pastoris*. Lane M, low molecular weight protein standards; lane 1, crude enzyme; lane 2, purified VfAly7; Figure S3: General scheme of the enzymatic approach for the utilization of brown seaweed (*U. pinnatifida*). The recoveries of alginates, proteins and minerals from brown seaweed (100 g) are indicated.
