# Supplementary material for: Releasing Bioactive Compounds from Brown Seaweed with Novel Cold-Adapted Alginate Lyase and Alcalase
Source: Mar Drugs. 2023 Mar 27;21(4):208. doi: 10.3390/md21040208 (PMC10142901; doi:10.3390/md21040208)
Supplement: Supplementary file 1 [file marinedrugs-21-00208-s001.zip › marinedrugs-2170976-supplementary.pdf]

Supplementary Materials

Releasing Bioactive Compounds from Brown Seaweed with Novel Cold-Adapted Alginate Lyase and Alcalase

Jun Jiang [1], Zhengqiang Jiang [1], Qiaojuan Yan [2], Susu Han [1] and Shaoqing Yang [1,*]

[1] College of Food Science and Nutritional Engineering, China Agricultural University, No.17 Qinghua East Road, Haidian District, Beijing 100083, China
[2] Key Laboratory of Food Bioengineering (China National Light Industry), College of Engineering, China Agricultural University, No.17 Qinghua East Road, Haidian District, Beijing 100083, China
* Correspondence: ysq@cau.edu.cn.

Table S1. Substrate Specificity of VfAly7.

Substrate	Specific activity (U/mg)	Relative activity (%)
sodium alginate	71.5	100%
poly MG	68.3	96%
poly M	47.2	66%
poly G	34.3	48%
Chitin	0	0
Agarose	0	0
mannan	0	0
CMC	0	0
cellulose	0	0

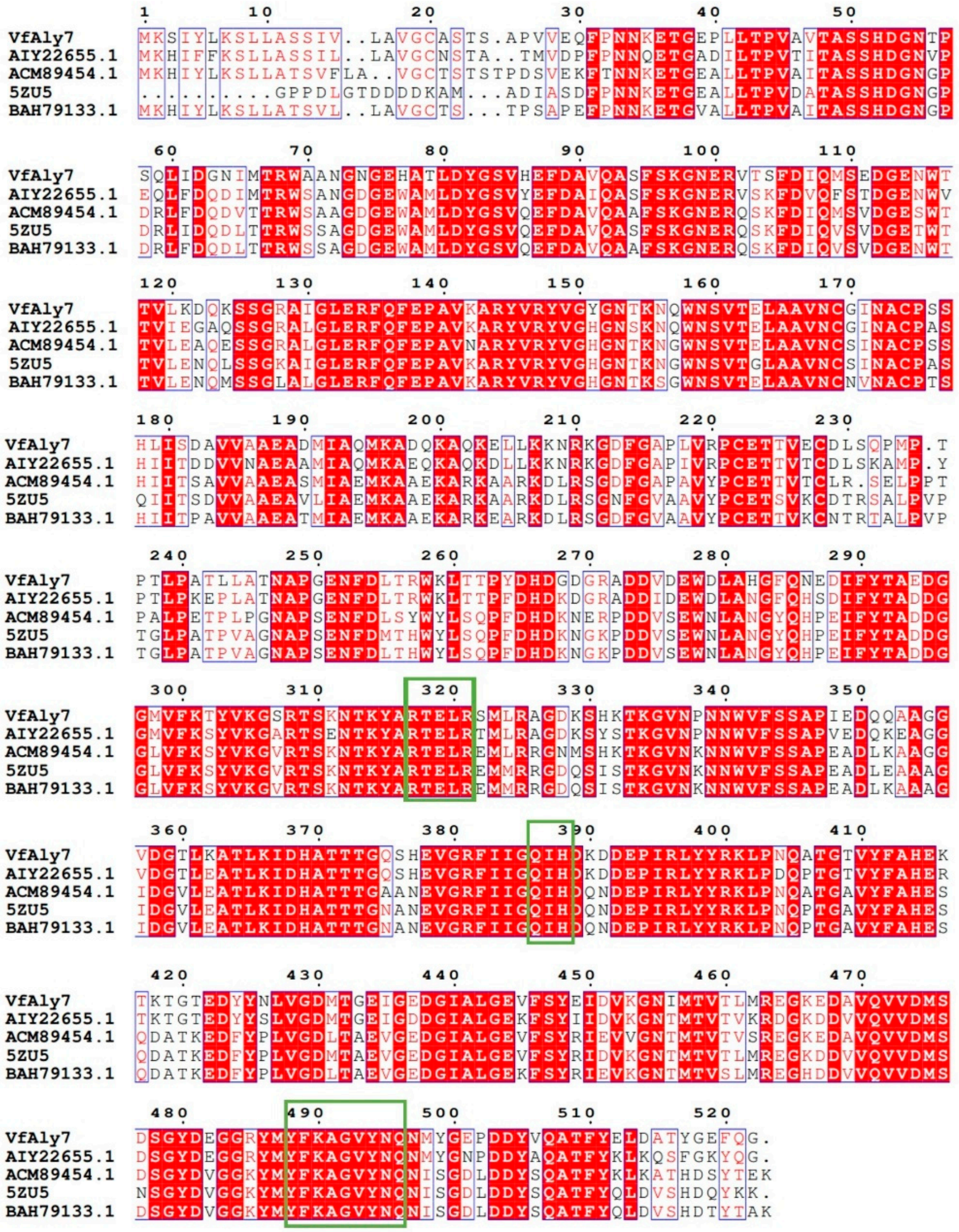

Figure S1. Multiple sequence alignment of VfAly7 and other well-characterized alginate lyases from PL family 7. Identical residues are shown in white on a red background, and conserved amino acid regions are marked in the green boxes.

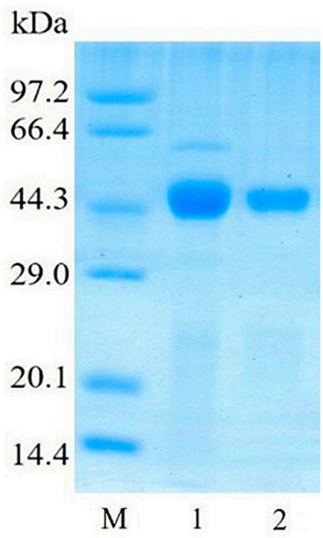

Figure S2. Purification of the recombinant VfAly7 expressed in *P. pastoris*. Lane M, low molecular weight protein standards; lane 1, crude enzyme; lane 2, purified VfAly7.

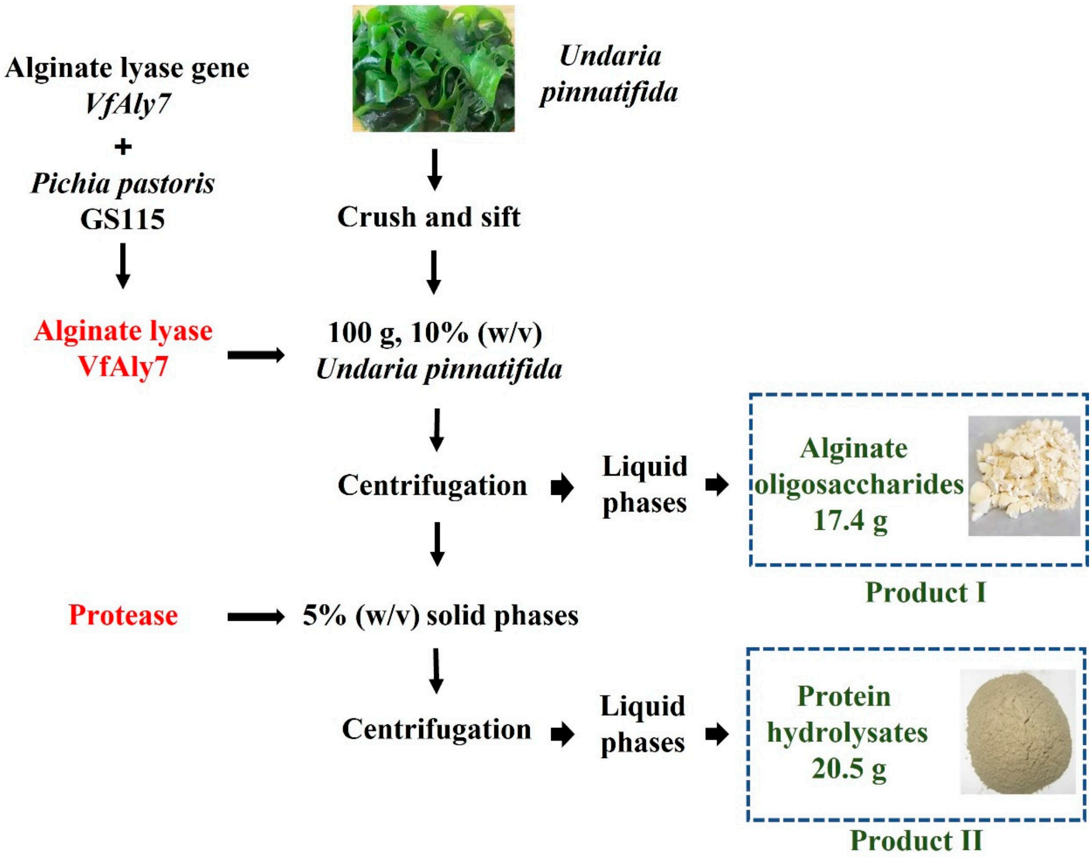

Figure S3. General scheme of the enzymatic approach for the utilization of brown seaweed (*Undaria pinnatifida*). The recoveries of alginates, proteins and minerals from brown seaweed (100 g) are indicated.
